# Supplementary material for: Acceptability and implementation potential of a health literacy intervention to increase colorectal cancer screening in deprived areas: A qualitative study of patients and general practitioners participating in a cluster randomized controlled trial
Source: PLoS One. 2025 Aug 5;20(8):e0329639. doi: 10.1371/journal.pone.0329639 (PMC12324091; doi:10.1371/journal.pone.0329639)
Supplement: Attachment — Submitted filename: Response to Reviewers.docx [file pone.0329639.s004.docx]

Alix BOIROT
SESSTIM UMR 1252
Aix-Marseille Université

Faculté de Médecine- 3e étage - Aile Bleue

27, boulevard Jean Moulin

13385 MARSEILLE cedex 5 – France

+33695590193

Alix.boirot@inserm.fr

**To the Editorial Board of *PLOS One***

19 March 2025

Dear Editors,

Thank you for your feedback on our original article entitled *“Acceptability and Implementation Potential of a Health Literacy Intervention to Increase Colorectal Cancer Screening in Deprived Areas”* and the opportunity to submit a revised draft of the manuscript to *Plos One.*

We responded point-by-point below to the reviewers’ comments, detailing changes made to the manuscript. These changes are highlighted in red in the revised version.

We hope these modifications adequately address the issues raised, and we remain at your disposal should further changes need to be made to the manuscript.

Yours faithfully,

Alix Boirot, on behalf of all the authors

**Journal Requirements**:

2. In the ethics statement in the Methods, you have specified that verbal consent was obtained. Please provide additional details regarding how this consent was documented and witnessed, and state whether this was approved by the IRB.

**Answer**:

We have added a paragraph explaining the Ethics approval in the method’s section, and we had already explained how GPs recruited patients with verbal consent required, which was approved by the Ethics committee in the section “Patient recruitment”: “As approved by CER, GPs obtained verbal consent from the patient, explained the study to them, and gave them a CRC self-test kit. In the intervention group, the GPs also used their training, the pictorial brochure, and the video to explain how to do the CRC screening test at home. All patients were followed up by telephone after 1 week and then 1 year to determine if they had taken the test (primary outcome) » We have added the following sentence for clarity (p.8, line 190) “At each interaction with a recruited patient, we checked they were still in agreement to consent, giving participants the opportunity to withdraw if they did not want to continue.”

3. Thank you for stating the following in the Competing Interests section: “I have read the journal's policy and one author of this manuscript have the following competing interests: MA-D has contributed to the development of Option Grid patient decision aids. EBSCO Information Cervices sells subscription access to Option Grid patient decision aids. She receives consulting income from EBSCO Health, and royalties. No other competing interests declared.”

Please confirm that this does not alter your adherence to all PLOS ONE policies on sharing data and materials, by including the following statement: "This does not alter our adherence to PLOS ONE policies on sharing data and materials.” (as detailed online in our guide for authors <http://journals.plos.org/plosone/s/competing-interests>). If there are restrictions on sharing of data and/or materials, please state these. Please note that we cannot proceed with consideration of your article until this information has been declared.

Please include your updated Competing Interests statement in your cover letter; we will change the online submission form on your behalf.

**Answer**: all these statements have been added: “We have read the journal's policy and one author of this manuscript has the following competing interests: Marie-Anne Durand has contributed to the development of Option Grid patient decision aids. EBSCO Information Cervices sells subscription access to Option Grid patient decision aids. She receives consulting income from EBSCO Health, and royalties. This does not alter our adherence to PLOS ONE policies on sharing data and materials. No other competing interests declared.”

4. We note that you have indicated that there are restrictions to data sharing for this study. For studies involving human research participant data or other sensitive data, we encourage authors to share de-identified or anonymized data. However, when data cannot be publicly shared for ethical reasons, we allow authors to make their data sets available upon request. For information on unacceptable data access restrictions, please see <http://journals.plos.org/plosone/s/data-availability#loc-unacceptable-data-access-restrictions>.

Before we proceed with your manuscript, please address the following prompts:

a) If there are ethical or legal restrictions on sharing a de-identified data set, please explain them in detail (e.g., data contain potentially identifying or sensitive patient information, data are owned by a third-party organization, etc.) and who has imposed them (e.g., a Research Ethics Committee or Institutional Review Board, etc.). Please also provide contact information for a data access committee, ethics committee, or other institutional body to which data requests may be sent.

b) If there are no restrictions, please upload the minimal anonymized data set necessary to replicate your study findings to a stable, public repository and provide us with the relevant URLs, DOIs, or accession numbers. Please see <http://www.bmj.com/content/340/bmj.c181.long> for guidelines on how to de-identify and prepare clinical data for publication. For a list of recommended repositories, please see <https://journals.plos.org/plosone/s/recommended-repositories>. You also have the option of uploading the data as Supporting Information files, but we would recommend depositing data directly to a data repository if possible.
**Answer:** As stated during the submission process, all data are available by request to the research team. Please contact either the corresponding author or the trial manager Dr Niamh M Redmond (niamh-maria.redmond@univ-tlse3.fr) for further information.

Please update your Data Availability statement in the submission form accordingly.

5. Please include your full ethics statement in the ‘Methods’ section of your manuscript file. In your statement, please include the full name of the IRB or ethics committee who approved or waived your study, as well as whether or not you obtained informed written or verbal consent. If consent was waived for your study, please include this information in your statement as well.

**Answer**: the ethics statement is now in the “methods” section

6. Please include captions for your Supporting Information files at the end of your manuscript, and update any in-text citations to match accordingly. Please see our Supporting Information guidelines for more information: <http://journals.plos.org/plosone/s/supporting-information>.

**Answer**: At the end of the manuscript there is now a statement about the list of supporting files available.

**Additional Editor Comments:**

Abstract

**Reviewer’s comments:**

1. Expand Background: Briefly introduce why colorectal cancer (CRC) screening is critical, particularly among socially disadvantaged populations.
2. Highlight Methodology: Specify the study design (qualitative, thematic analysis) and how participants were recruited.
3. Clarify Key Findings: Clearly state the level of preference for the video versus the brochure (e.g., percentages or qualitative themes).

**Response:**

- Point 1 : We had already addressed this in the abstract: “In France, despite a free organised screening programme for people aged between 50 and 74, participation rates remain suboptimal. Socioeconomic position and health literacy levels exacerbate the situation, with the lowest screening rates observed in the most socially disadvantaged areas of the country.”
- Point 2: We had already stated this in the abstract (please see bold wording). We added “in the DECODE project” in the abstract for clarity (p.2, line 46):

“We conducted a **cross-sectional qualitative study** using semi-structured interviews with patients (n=24) and GPs (n=22) **who used or participated** in the DECODE project **intervention.** The interviews were conducted by telephone or videoconference, and were analysed thematically using Nvivo software and dual independent coding.”

- Point 3: The result section of the abstract has been rearranged in order to highlight the findings (p.2, line 50):

“95% of GPs expressed a clear preference for the video over the brochure. Patients had varied results with 50% preferring the video, as it demonstrated how to do the test, versus the brochure. The humorous and de-dramatising aspects of the video were the two key factors highlighted by interviewees. However, support from healthcare staff (GPs, nurses, etc.) is still essential, in supporting patients in prevention. This presents a challenge for GPs, who are frequently constrained by time limitations during consultations.”

**Reviewer’s comments:**

4. Strengthen the Conclusion: Suggest how findings may be translated into policy or practice, particularly in improving screening uptake.
**Response:**

- We have revised the abstract's conclusion to highlight how our findings can inform policy and practice, particularly in improving CRC screening uptake (p.2, lines 59-62):

“Our findings emphasize the need to tailor promotional materials for both patients and healthcare professionals to improve CCR screening uptake, balancing digital efficiency with maintaining core human relationships in healthcare. Such intervention can be integrated into different workflows. The addition of video into national CRC screening programs might also help. Targeting CRC screening interventions at provider-patient interactions, ensuring they are tailored, accessible, and engaging, is key to reducing disparities.

Introduction

**Reviewer’s comments:**

1. Define the Problem Clearly: Provide more statistics on CRC incidence, mortality, and screening uptake disparities to establish the study’s significance.

**Response:**

- The introduction has been expanded with additional references supporting our arguments (p.3, lines 71-76):

“According to the World Health Organization (WHO), since 2020, 1.8 million new cases of colorectal cancer have been recorded worldwide, making it the third most common cancer after lung and breast cancer, and the second leading cause of cancer deaths. This disease predominantly affects Western countries, with particularly high incidence rates observed in Europe, Australia and New Zealand (4).”

**Reviewer’s comments:**

2. Link to Health Equity: Discuss the social determinants of health and their role in screening participation, incorporating health literacy and digital access considerations.

**Response:**

- The introduction has been expanded with additional references arguing our statements (p.4, lines 86-96):

“Since the implementation of the national CRC screening programme, participation rates have remained unchanged despite campaigns to increase accessibility to CRC screening kits - available for free in pharmacies or directly online at [ameli.fr](http://ameli.fr) (12). A recent study (13) demonstrated that in France the most disadvantaged populations (based on European Deprivation Index (EDI) data) had a lower net 5-year survival rate for most cancers and in particular for CRC. These findings align with international data (14). Additionally, social inequalities in health affect the most disadvantaged populations both in terms of higher exposure to risk factors and reduced access to care (15). As well as socioeconomic position, lower health literacy is associated with poor health outcomes, poorer overall health status, higher mortality rates (16), and less engagement with prevention services including CRC screening (17).”

**Reviewer’s comments:**

3. Intervention Justification: Elaborate on why video interventions might be more effective than brochures, citing relevant behavioral science theories.

**Response:**

- Our study does not aim to compare the effectiveness of video versus brochure interventions; rather, it examines participants' preferences regarding these formats.

**Reviewer’s comments:**

4. Hypothesis or Study Objectives: Clearly state the research questions or hypotheses guiding the study.

**Response:**

- Hypothesis have been enhanced (p4, lines 104-107):

“The involvement of general practitioners (GPs) is essential, as they serve as the primary point of contact for many people. Our hypothesis is that the manner in which the importance of the CRC test is introduced and presented, using clear language plays a crucial role in screening rates, particularly among the most disadvantaged populations.”

Methods

**Reviewer’s comments:**

1. Clarify Inclusion/Exclusion Criteria: Define how patients were selected, specifying characteristics such as age, previous screening history, or language barriers.

**Response:**

- The inclusion/exclusion criteria were already explained in the methods section under “Patient recruitment, for more clarity we added some details (p.7, line 171):

(i.e. did not conduct a CRC screening test within the previous two years)

**Reviewer’s comments:**

2. Expand on GP Recruitment: Explain how GP practices were approached and how many declined participation.

**Response:**

- A more detailed explanation of GP recruitment has been added (p7, lines 162-164):

“GPs were contacted by email or telephone by the research team and follow-up if necessary. We used our research and community contacts to reach other GPs via ‘snowballing’ to invite other GPs to participate”.

- We do not have data on how many declined as many just did not respond to emails or telephone calls.

**Reviewer’s comments:**

3. Interview Protocol: Provide more details on the interview guide—what key questions were asked, and how responses were probed for depth?

**Response:**

- We have added a section that explains the key questions from the interviews (p9 lines 215-218):

“In particular, the question posed to the GP in the interview guide/GP “what did your patients think of the brochure and the video? “ (see Appendix 1) and the questions posed to the patients in the patient interview guide “What do you think of the brochure?“ and “What do you think of the video?“ (see Appendix 1) allowed us to identify any differences or similarities in perceptions regarding the information tools.”

**Reviewer’s comments:**

4. Data Management: Describe how qualitative data were transcribed, coded, and validated (e.g., was double-coding used for reliability?).

**Response:**

- This information has already been reported in the section 'Data analysis' data analysis”, however for more clarity we add “each interview was double-coded” before the procedure explanation p9 line 224.

**Reviewer’s comments:**

5. Ethical Considerations: Clarify how participant confidentiality was ensured and whether incentives were provided.

**Response:**

- We added a statement in order to describe more in details how confidentiality was ensured (p9, lines 221-222):

The transcripts were anonymised, with only the socio-demographic (age, gender) and literacy data (for patients) linked to them.

Results

**Reviewer’s comment:**

*1. Demographic Breakdown: Include a table summarizing participant characteristics (e.g., age, gender, education, previous screening history).*

**Response:**

- This information was already included in the original submission. To ensure clarity, we have reformatted the tables for improved readability (Tables 1 and 2 in the revised manuscript). The data concerning previous screening history was not available.

**Reviewer’s comment:**

*2. Thematic Structure: Clearly separate results by themes, ensuring that GP and patient perspectives are distinct.*

**Response:**

- We have added thematic subsections in the results section to clarify the presentation of the results as suggested:

2. Patients and GPs prefer video over brochure for clarity, humor, and effective messaging

a) Video: A Pedagogical and Engaging Tool

b) Humor as a tool to normalize CRC screening

c) Brochure: underutilization and misunderstanding

d) A preference for the video over the brochure

3.  Integration of the DECODE intervention into primary care consultations: duration, challenges, and patient preferences

 a) Consultation context and time constraints

 b) Time as a necessary investment and a key barrier

c) Using the video to optimize time

4. Improve prevention by disseminating tools and delegating preventive tasks to meet GPs’ time constraints and patient preferences

a) Delegation of preventive tasks to other healthcare professionals

b) Expanding awareness and increasing accessibility of CRC screening

- Additionally, we included introductory and transition sentences to enhance clarity and ensure a clear distinction between patient and GP perspectives (p16, lines 285-290):

“The video and brochure, both designed to promote CRC screening, were perceived by patients and GPs as serving different roles. The brochure was viewed as a useful reminder for patients to use at home, while the video was regarded as a tutorial-style tool. However, while the video was valued for its clarity and engaging approach, the brochure was often underutilized, with some GPs finding it redundant. This section explores how each tool was received and the reasons behind the clear preference for the video.

- As the aim of this paper is to carry out a cross-analysis of the points of view of patients and GPs, we have not separated them by section. However, we have ensured that the separation of the points of view of patients and GPs is clearer by adding transitional sentences:

This perception was shared by GPs, who noted that humor helped reduce patient stress and make the screening process feel less intimidating. Several GPs reported that patients often laughed or smiled while watching the video, which created a more relaxed atmosphere during consultations.” (p17, line 321-324)

“From the patients’ perspective, the brochure's content was still considered too dense, which tended to water down the key messages.” (p18, line 341)

“Even though GPs faced these challenges, patients still expressed a clear preference for receiving the DECODE brochure and video during consultations with their GP or another healthcare professional, valuing the opportunity for discussion and finding the presence of a healthcare professional reassuring and valuable.” (p21, line 397)

“This sense of direct engagement with a healthcare professional was particularly important for patients who found that a conversation helped simplify complex information and ease concerns about screening.” (p21, lines 405-407)

**Reviewer’s comment:**

*3. Direct Quotes for Depth: Incorporate more illustrative patient and GP quotes to support key themes.*

**Response:**

- We have added several patient quotes to better support our findings:

[…] the brochure's content was still considered too dense, which tended to water down the key messages:

*"...you can't say it's poorly done, but I find it packed with a lot, a lot of information. For example, I preferred the video a hundred times over this" Patient (14)*

Several misunderstandings were also raised, such as the images of the colon and intestine not being explicit enough, or words that were not understood, such as "literacy", misunderstood by at least two respondents:

*“Everything is complicated, the terms are complicated, so there you go, we really need a lot more diagrams and bigger diagrams.” Patient (13) (p18-19, lines 343-349)*

*“I had already had it in my hand [...] I think a year ago, as my mum had just had a positive test result [...] she was really worried [...] we had looked at it together, I had tried to reassure her a little “ Patient (14).*

*“Oh yes, of course (regarding sharing the brochure) [...] if, for example, at work, someone was to bring up the subject with me [...] I would easily be able to tell them that there are tools that are very well suited for this and that they can check out.” Patient (12).” (p.19, lines 353-358)*

**Reviewer’s comment:**

*4. Unexpected Findings: Discuss any surprising responses, such as participants who still preferred brochures despite the majority favoring videos.*

**Response:**

- The most striking finding was that the majority of patients expressed a negative view of the brochure, even though it had been designed specifically for the trial for and by people with low literacy. This observation has been previously addressed in the paper; however, additional elements have been incorporated to provide a more comprehensive explanation of the patients' feedback on the brochure:

“However, among the respondents, 10 said they had used the DECODE brochure to talk about colorectal cancer screening with those around them (family, colleagues), and 13 are planning to do so:

“I had already had it in my hand [...] I think a year ago, as my mum had just had a positive test result [...] she was really worried [...] we had looked at it together, I had tried to reassure her a little “ Patient (14).

“Oh yes, of course (regarding sharing the brochure) [...] if, for example, at work, someone was to bring up the subject with me [...] I would easily be able to tell them that there are tools that are very well suited for this and that they can check out.’ Patient (12).“ (p19, lines 350-358)

“(...) by reading, I find it much less accessible than video” - Patient (6) (p.19, line 364)

**Reviewer’s comment:**

*5. Barriers to Implementation: Describe any challenges participants mentioned in accessing or engaging with the intervention.*

**Response:**

- We had already discussed key barriers such as time constraints, underutilization of materials, digital literacy issues, and cultural inclusivity. To enhance clarity, we have added a sub-section title (p22, line 412):

“b) Time as a necessary investment and a key barrier”

along with a sentence emphasizing GP time constraints (p23, lines 459-461):

“However, they also acknowledged that, in a busy practice, time constraints often made it difficult to consistently engage in detailed discussions, forcing them to prioritize certain aspects of care over others.”

Discussion

**Reviewer’s comment:**

*1. Compare to Existing Literature: Situate findings within the broader research on CRC screening interventions, highlighting similarities and differences.*

**Response:**

- As our study focuses on acceptability rather than effectiveness (which will be the subject of the primary trial publications), we have limited direct comparisons. In addition to the papers placing this intervention among health interventions using videos and/or brochures in general, we have added a reference to a relevant CRC screening study (p27, line 551-552):

“This aligns with previous findings showing that educational videos increase knowledge and reduce negative attitudes towards CRC screening (38).

**Reviewer’s comment:**

*2. Behavior Change Mechanisms: Explore why the video may have been more effective, referencing behavior change theories (e.g., Health Belief Model, Theory of Planned Behavior).*

**Response:**

- We have integrated the Health Belief Model (HBM) into our discussion (p27 lines 543-552):

“Patients were able to clearly understand the advantages and benefits of doing the CRC screening test, dispelling fears and uncertainties. According to Krouse (35), using video in clinical practice has benefits in assisting with decision-making and reducing anxiety. According to the Health Belief Model (HBM) (36,37), individuals are more likely to engage in preventive health behaviors when they perceive a serious health threat, recognize the benefits of taking action, and feel that barriers to action are minimal. In our study, the video appeared to address key perceived barriers to CRC screening by providing a clear, step-by-step demonstration of the test, which likely increased self-efficacy—a crucial determinant of health behavior adoption. Additionally, by normalizing the test through humor, the video may have reduced anxiety and embarrassment, which are often cited as psychological barriers to CRC screening. This aligns with previous findings showing that educational videos increase knowledge and reduce negative attitudes towards CRC screening (38).

**Reviewer’s comment:**

*3. GP Workload Constraints: Discuss how time constraints among GPs might limit intervention feasibility and suggest strategies to overcome this*.

**Response:**

- We have already provided this information in the original submission. However, we have added two paragraphs clarifying this point (p29, lines 593-603) (p30, lines 619-621):

“Despite the overall acceptability of the intervention, barriers to implementation were identified. Interviews with GPs revealed that the main barrier they faced in introducing screening for CRC was a lack of time. GPs reported that time constraints often led to screening discussions being deprioritized in favor of more immediate medical concerns, resulting in low engagement with the intervention tools. Some GPs admitted that, due to time pressure, they simply handed the brochure to patients without explaining its content, significantly reducing its effectiveness. To facilitate the normalization of this intervention and to address GPs’ time constraints while honoring patient preferences, new organizational workflows are needed. While the implementation of video displays in GP waiting rooms could be regarded as a time-saving strategy, research indicates that while it may enhance patients' knowledge, it may not be effective in modifying health behaviors (47).

“One GP noted that nurses, trained in motivational interviewing, achieved impressive success rates for screenings, largely due to the time spent on consultations. Indeed, several studies have demonstrated that motivational interviewing has the potential to enhance the uptake of health screenings (49). While delegating the task to nurses or medical assistants has shown promising results, this approach requires sufficient staffing and adequate training, which may not be available in all healthcare settings.”

**Reviewer’s comment:**

*4. Digital Divide Issues: Acknowledge potential limitations related to digital literacy and access to video interventions in disadvantaged populations.*

**Response:**

- We added a paragraph discussing digital literacy challenges (p28, lines 573-580):

“Moreover, it is of the utmost importance that cultural sensitivity be incorporated into the development and delivery of such videos (42). Older patients (≥65 years old) and individuals from low socioeconomic backgrounds often encounter difficulties using digital (43) devices which can act as a significant barrier to health interventions (44). This is why, although integrating the video into the waiting room or providing a QR code on the brochure could be seen as a time-saving solution for GPs, such an approach would not be suitable for our target population- which includes elderly and socioeconomically disadvantaged patients. Additionally, patients themselves expressed a strong preference for face-to-face interactions over digital-only solutions. The majority of patients expressed a greater preference for watching the video within the GP practice, underscoring the importance of patient-provider communication and the value of personalized interactions in promoting adherence to CRC screening recommendations.”

**Reviewer’s comment:**

*5. Cost-effectiveness Consideration: Briefly discuss whether video interventions are cost-effective compared to traditional methods like printed brochures.*

**Response:**

- The purpose of this paper was to study the acceptability of such an intervention for GPs and patients. The question of cost will be addressed in other publications concerning the trial. However, it should be noted that even if the production of a video can be expensive, its cost becomes more negligible when it is aimed at several million people.

Limitations

**Reviewer’s comment:**

*1. Potential Biases: Address possible social desirability bias in participant responses.*

**Response:**

We have added a paragraph acknowledging this bias (p.30, lines 628-633): “It is possible that the participants' responses were influenced by social desirability bias. The participants may have been inclined to provide answers perceived as socially acceptable, particularly to express more favorable opinions towards the video and brochure than they would have in an informal setting. However, it should be noted that the patients interviewed were quite capable of criticising the brochure produced by the research team, which goes against a very strong social desirability bias.

**Reviewer’s comment:**

*2. Generalisability: Acknowledge that findings may not apply to other healthcare settings or more affluent populations.*

**Response:**

- We have added the following sentence (p31, lines 642-643):

“The nested nature of the study within a trial targeting underserved areas also implies that the sample may not be wholly representative of all GPs or patients. Specifically, just few single-handed GPs were included and they have withdrawn from the study, and most participants operated within multidisciplinary practices. This could limit the generalizability of our findings, as single-handed GPs may face different constraints and working conditions that were not captured in our analysis.”

**Reviewer’s comment:**

3*. Short-Term vs. Long-Term Impact: Mention that the study focused on acceptability but did not measure long-term changes in screening behavior.*

**Response:**

- We have clarified this limitation and noted that long-term impact will be assessed in the primary trial (p31, lines 645-649):

“Furthermore, there was some confusion among respondents regarding which brochure was being referred to, which could have impacted the clarity and consistency of the responses. In addition, while this study focused on the acceptability of the intervention, it did not assess long-term changes in screening behavior. However, this limitation will be addressed in the primary trial, which was designed to evaluate the actual impact of the intervention on screening uptake up to a year after the recruitment consultation.”

**Reviewer’s comment:**

4. Self-Selection Bias: Discuss whether more health-conscious individuals may have been more likely to participate.

**Response:**

- We have added the following paragraph highlighting the potential biases of self-selection (p31, lines 634-636):

“A self-selection bias may also have influenced our results. Patients and GPs who agreed to take part in the study are potentially more aware of prevention and screening issues than those who refused.”

Conclusion

**Reviewer’s comment:**

*1. Summarize Practical Takeaways: Provide clear recommendations for implementing video-based interventions in primary care.*

**Response:**

- We have added a paragraph highlighting in the conclusion structured workflows and task delegation to optimize feasibility (p32, lines 668-674):

“It highlights the importance of striking a balance between the efficiency of digital tools and maintaining good and trusting relationships at the core of healthcare. From a practical perspective, our study suggests that video-based interventions could be integrated into primary care settings in a way that respects both the time constraints of GPs and the preferences of patients for in-person communication. To maximize feasibility, healthcare providers could implement structured workflows, such as showing the video during consultations while doing administrative tasks or delegating the screening promotion task to advanced nurse practitioners or trained healthcare assistants, who have demonstrated success in patient engagement.”

**Reviewer’s comment:**

2. *Call for Future Research: Suggest the need for a follow-up study assessing actual screening uptake after exposure to the intervention.*

**Response:**

- The primary trial is designed to evaluate the actual impact of the intervention on screening uptake up to 1 year after the recruitment consultation. The results of this trial will soon provide an evaluation of the intervention's efficacy. We added two sentences to clarify it in the discussion (p27, line 531):

“The objective of this qualitative study was to assess the acceptability and feasibility of a HL intervention designed to enhance CRC screening in disadvantaged areas with regard to both GPs and patients. The primary trial will evaluate the impact of the intervention.”

- In the conclusion (p33, lines 683-684) :

“In addition to those results, the primary trial (NCT04631692) will evaluate the actual impact of the intervention on screening uptake up to 1 year after the recruitment consultation.”

**Reviewer’s comment:**

3*. Policy Implications: Discuss how health systems can integrate video interventions into routine CRC screening promotion strategies.*

**Response:**

- We have included a sentence on potential integration into national CRC screening programs (p32, lines 675-676):

“At a policy level, integrating this video into national CRC screening programs could be an effective way to improve adherence rates, particularly in disadvantaged communities.”

**Reviewer’s comment:**

*4. Final Thought: End with a strong statement on the importance of tailored interventions for improving screening rates among disadvantaged groups.*

**Response:**

- We added a concluding paragraph emphasizing accessibility, engagement, and the role of healthcare professionals (p33, lines 685-689):

“Ensuring that CRC screening interventions are tailored, accessible, and engaging is critical to addressing disparities in screening participation. By leveraging audiovisual tools, humor, and trusted healthcare professionals, health systems can empower patients, reduce screening hesitancy, and ultimately improve early detection and outcomes for colorectal cancer, particularly among disadvantaged populations.”

Additional Recommendations for Tables & Figures

**Reviewer’s comment:**

1. Comparison Table: Include a table comparing the key themes between GP and patient responses.

**Response:**

- We added a table comparing the key themes between GP and patient’s responses:

Comparison of GP vs. Patient Opinions

| **Themes** | **GP’ opinions** | **Patients’ opinion** |
| --- | --- | --- |
| **Use of the video** | Video considered highly educational and accessible, particularly for patients with reading difficulties  Appreciated for making the topic less daunting, facilitating test acceptance | Video perceived as clear, simplifying the test process  Humor helps reduce anxiety, making screening more approachable |
| **Use of the brochure** | Often underutilized as it is perceived as redundant | Brochure seen as dense, sometimes misunderstood, but useful for sharing with relatives |
| **Time constraints** | Lack of time during consultations, difficulty in integrating an in-depth discussion on screening | Patients appreciate the time GPs dedicate to explanations |
| **Preference for distribution setting of tools** | Prefer distribution during consultations but sometimes used in the waiting room | Prefer distribution during consultations, with physician guidance |
| **Suggestions for improving implementation** | Proposal to delegate prevention to nurses or medical assistants | Suggestion to broadcast the video in waiting rooms and on social media |

**Reviewer’s comment:**

2. Flowchart: If applicable, provide a flowchart of participant recruitment and data collection.

**Response:**

- A flowchart is not applicable for this type of qualitative study. The recruitment process and data collection are straightforward and clearly described in the methods section.

**Reviewer’s comment:**

3. Graphical Abstract: Consider adding a simple visual summary of the study’s main findings for better accessibility.
**Response:**

- Thank you for this suggestion. While we acknowledge the potential value of a graphical abstract, we believe that the key findings are already well-summarized in the abstract and main text. Given the scope and focus of our study, we have opted not to include a graphical abstract at this time.
